# Supplementary figures and images for: Chromosome-level genome of Schistosoma haematobium underpins genome-wide explorations of molecular variation
Source: PLoS Pathog. 2022 Feb 15;18(2):e1010288. doi: 10.1371/journal.ppat.1010288 (PMC8846543; doi:10.1371/journal.ppat.1010288)

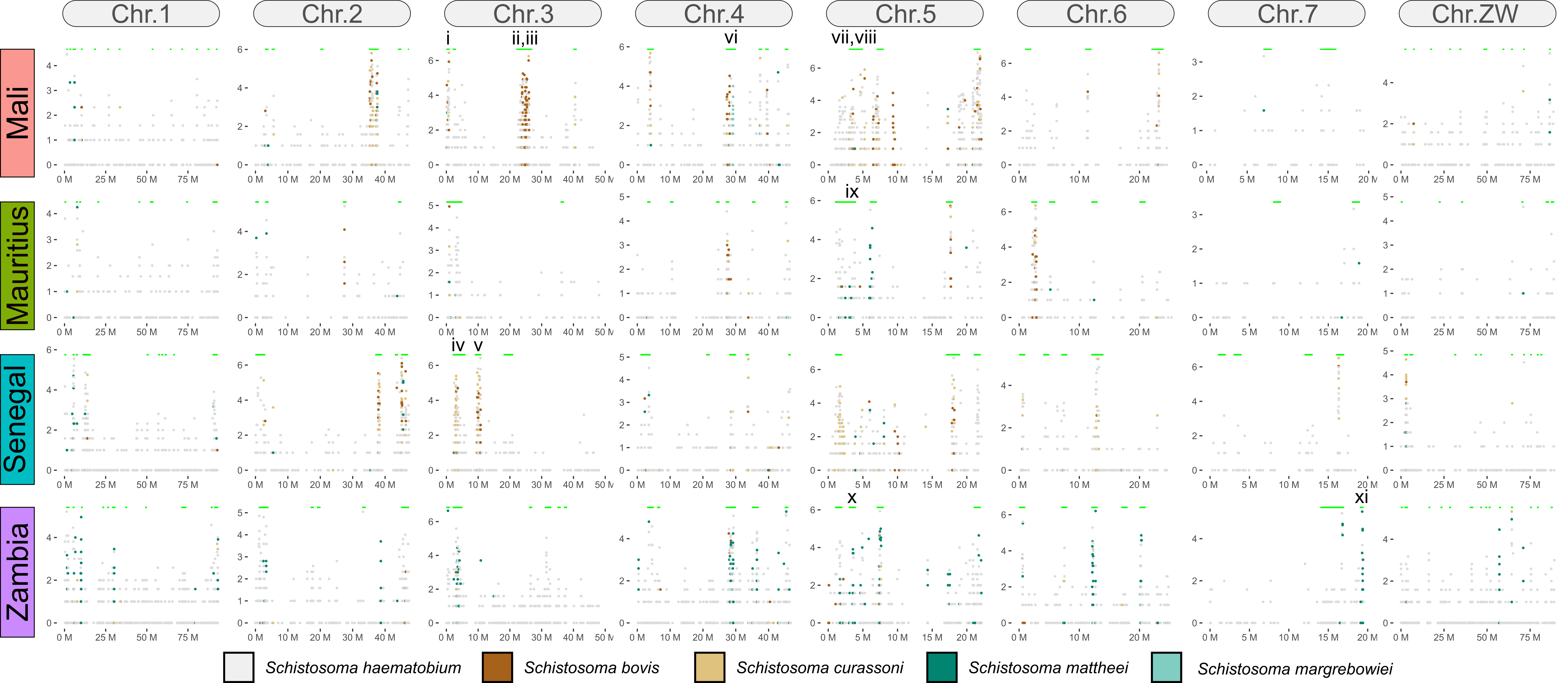

Supplement: S1 Fig — For isolates from Zambia, Senegal, Mauritius or Mali, density and localisation of SNPs in the S. haematobium reference genome are shown in 2 kb non-overlapping regions, with each point coloured by the species with the closest nucleotide sequence homology. For each sample, SNP-rich regions (light green blocks) of which > 20% resembled a genomic reference other than S. haematobium are labelled (i-xi). (TIFF) [file ppat.1010288.s001.tiff]
