# Supplementary material for: Chromosome-level genome of Schistosoma haematobium underpins genome-wide explorations of molecular variation
Source: PLoS Pathog. 2022 Feb 15;18(2):e1010288. doi: 10.1371/journal.ppat.1010288 (PMC8846543; doi:10.1371/journal.ppat.1010288)
Supplement: Attachment — Submitted filename: Rejoinder.ShaeV3. R1.docx [file ppat.1010288.s020.docx]

Professors Haldar and Malim

Editors-in-Chief for *PLoS Pathogens*

11 January 2022

**Dear Professors Haldar and Malim,**

**RE:** **Manuscript PPATHOGENS-D-21-02181.R1**

We submit our revised manuscript entitled “Chromosome-level genome of *Schistosoma haematobium* underpins genome-wide explorations of molecular variation” for publication in *PLoS Pathogens*.

**We thank you and the three reviewers for their positive and constructive reports on our manuscript. We are pleased that all three reviewers believe that the present work represents an important advance and useful resource for the research community, and that it fits the scope of *PLoS Pathogens*.**

**The present rejoinder addresses all of the reviewers’ suggestions and recommendations (responses in bold-type) in a point-by-point manner:**

**REVIEWER #1:**

**1.1.** In the section of Introduction: “[…] drug can sometimes be ineffective 9-11, and treatment alone does not achieve sustainable control of this disease in endemic countries {Knopp, 2019; #6919;Aula, 2021 #6575}. The drug may be ineffective for juvenile schistosomes, but I do not understand what “Sometimes” means in the sentence and also have no idea about the information in parentheses { }.

**Response 1.1. We have modified the sentence to address this issue (lines 59-60): “However, treatment efficacy with this drug can be variable, such that mass treatment alone might not achieve a sustainable control of this disease [9-13].” We have elected to cite two additional references:**

**12.	Aula OP, McManus DP, Jones MK, Gordon CA. Schistosomiasis with a focus on Africa. Trop Med Infect Dis. 2021;6(3). https://doi.org/10.3390/tropicalmed6030109 PMID: 34206495**

**13.	Knopp S, Ame SM, Person B, Hattendorf J, Rabone M, Juma S, et al. A 5-Year intervention study on elimination of urogenital schistosomiasis in Zanzibar: Parasitological results of annual cross-sectional surveys. PLoS Negl Trop Dis. 2019;13(5):e0007268. https://doi.org/10.1371/journal.pntd.0007268 PMID: 31059495**

**1.2.** Three versions of *S. haematobium* were generated from the same group in collaboration with different investigators. I understand that the third version (chromosome-level assembly) was produced using a combination of Hi-C sequencing and other data. It is unclear which data are unpublished or new and which were published or used in the previous publications. Also, in the section of Result, it stated that Hi-C and long-read data were used. In the section of Materials and Methods, it said short Illumina reads were also used.

**Response 1.2. Much of the data produced here are new and have been deposited in the Sequence Read Archive under accession numbers SRR15400745 to SRR15400772 (Data Availability statement). In the revised manuscript, we have now included Supplementary Table 1, which provides detail on the new datasets. In the text, we also describe other data sets produced in previous studies (now refs 25 and 26) (lines 326 - 329) and how they were used (lines 330 - 338).**

**1.3.** In the section of results: A comparison of genome Shae.V3 with a previous assembly for *S. haematobium* (i.e. Shae.V2 with 130 scaffolds) (Table 1; Fig. 1d) showed that Shae.V3 is substantially more contiguous. I expected Shae V3 should have a smaller number of scaffolds as compared to Shae V2. Here it said the number of scaffolds of Shae.V2 is 130 whereas in Shae.V3, the number is 163. Table 1 however indicates Shae.V2 has 666 scaffolds. Please clarify it.

**Response 1.3. To clarify, Shae.V2 has 666 scaffolds, and Shae.V3 has 163 scaffolds (Table 1) (lines 87 - 88). Regarding contiguity, eight scaffolds contain 98% of the Shae.V3 assembly (line 88), linked to 130 scaffolds (97%) representing assembly Shae.V2 (Figure 1d) (lines 95 - 97).**

**1.4.** A number of genomes from different *Schistosoma* species are compared. It is unclear which version of genome for a given species are used in Table 1 as some species have more than one published version of genome sequences.

**Response 1.4. We obtained the data sets for all other schistosome species from WormBase ParaSite (release WBPS15). To clarify, we have added this information to Tables 1 and 2, in addition to the information described in the text (Table 1: line 816; Table 2: line 821).**

**1.5.** It is interest to know what re-arrangements are, although Supplementary Table 2 provides such information. Using diagrams to illustrate the re-arrangements is recommended.

**Response 1.5. We agree. The rearrangements are depicted in Figures 1b and 1d, and, as the reviewer indicates, the information is presented in Supplementary Table 2.**

**1.6.** This is a genome-level assembly. In theory, it should be assembled from (sic) 8 scaffolds. It is understandable that repeated sequences make it difficult to assemble chromosome scale scaffolds. But I think that the first 8 big scaffolds should represent most majority of the genome (i.e., 8 chromosomes). Such important information is not described in the paper.

**Response 1.6. We wish to refer to the Results section (lines 87 - 89) which states that 98% of the present assembly is represented by eight chromosomes. In addition, in Table 1, we provide the L90 metric (i.e. the number of scaffolds representing 90% of the assembly; L90 = 7) for Shae.V3.**

**1.7.** For the BUSCO analysis, it stated that the number of core genes within the Eukaryota dataset in Table 1 is 255, but the number in Table 2 is 954. I assume 954 is for Metazoa, which however is also different from Eukaryota.

**Response 1.7. We have added a footnote to Table 2 in the revised manuscript (line 820). To clarify, for the BUSCO analysis in genome mode (Table 1), we used the eukaryotic data set (255 BUSCOs), and for the analysis in protein mode (Table 2), we used the metazoan data set (954 BUSCOs).**

**1.8.** How to distinguish fixed SNPs (i.e., homozygous) from non-fixed SNPs?

**Response 1.8. Homozygous (or fixed) SNPs are those that are unambiguously different at individual positions from the *S. haematobium* reference genome. They were inferred using mapped genomic reads from individual male worms using GATK HaplotypeCaller, as described in the Methods (lines 448 - 462).**

**1.9.** RNAseq: Description of materials and methods is incomplete and unclear throughout the paper. The description was emphasized on how to use different softwares. How to properly handle biological materials is critical for obtaining reliable RNAseq data for subsequent analyses because gene expression is affected by many environmental factors, such as temperature of preserving or transporting samples, degradation of tissues or RNA samples, etc.

**Response 1.9. We respectfully disagree that the methodology is incomplete and unclear. We describe in detail how materials were processed, RNA isolated, handled and stored, how high RNA quality was assessed and how RNA-Seq data sets were produced (lines 361 - 376).**

**1.10.** Also, the data showed expression of the two sexes, but the materials said 50 worm pairs, in addition single sex samples, were used. Following questions should be clearly addressed in the paper. What biological replicates and/or technical replicates are for each sample? Where and how RNA extraction (for example, eggs from human urines) were done? How to transfer live tissue samples or RNAs to lab, etc.

**Response 1.10. We have stated clearly for which samples biological replicates were available (lines 361 - 364) and how RNA was extracted from individual samples (lines 363 - 365). We now include Supplementary Table 1, which describes all of the data sets produced in this study (including pooled samples and samples with biological replicates).**

**_______________**

**REVIEWER #2:**

**2.1.** Abstract: It is quite background-heavy and doesn’t focus much at all on what was done and what was found. These two aspects could be expanded on.

**Response 2.1. We agree. We have better described the findings and interpretations (lines 20 - 27).**

**2.2.** Introduction: Check the reference formatting. I.e. {Knopp, 2019 #6919;Aula, 2021 #6575}.

**Response 2.2. Thank you. We have addressed this in the revised manuscript (lines 60 - 61).**

**2.3.** “these studies usually…limited number of nuclear genetic markers” – perhaps this is a little unfair on the recent use of genomics involving *S. haematobium*, for example:

- Platt et al 2019 MBE

- Oey et al 2019 PLoS Pathogens

- Rey et al 2021 PLoS Pathogens – you do cite this, but before you state the limitation of no genomics

**Response 2.3. We have amended this section (lines 63-70) to reflect that studies on genetic variation of *S. haematobium* have historically relied mainly on a limited number of genetic markers and that although some genome-wide studies are now becoming available, they are still limited in numbers within the published literature.**

**2.4.** Results, reference genome: I like Figure 1. It could be helpful to mention the syntenic blocks are five or more single copy orthologs.

**Response 2.4. Thanks. We do mention syntenic blocks; the information is provided both in the legend of Figure 1 (“Syntenic blocks containing five or more single-copy orthologs (SCOs) between *S. haematobium* and the respective other species are shown as ‘links’”) and in the Methods (“with ≥ 5 SCOs per bundle”) (lines 356-359). Thus, no amendment is required.**

**2.5.** Following on, differences in block length are not obvious at the scale presented on the CIRCOS plots. Are you able to comment on the distribution of block lengths? Are there parts of genomes that are more conserved, relative to other regions, based on conservation of block length?

**Response 2.5. Supplementary Table 2 provides information of individual block lengths for each of the pairwise comparisons. In addition, we have now added the number of SCOs for each block and the difference in block size between the two species in the revised Supplementary Table 2.**

**2.6.** Figure shows “ZW chromosome” – these are obviously two chromosomes – Z and W - not one as indicated. Were you able to separate & define them in the assembly? This deserves some mention, especially given the efforts to do this by Buddenborg et al 2021 bioRxiv for *S. mansoni*.

**Response 2.6. The nomenclature of the sex chromosome(s) is consistent with previous publications for *S. mansoni*. Since the Z and W chromosomes of *S. haematobium* are not fully resolved (as was the case for *S. mansoni*), the designation “ZW” is appropriate for the large scaffold that aligns with the “ZW” scaffold (WBPS15) of *S. mansoni*. The study undertaken by Buddenborg et al. (2021 bioRxiv, ref. 39) for *S. mansoni* is exceptional. Using this unpublished work to guide sex chromosome assembly in *S. haematobium* is not appropriate, as this work has not yet undergone peer review and one cannot assume that there is no structural difference in the sex chromosomes between these species. Although the sex chromosomes of *S. haematobium* could not be unequivocally defined using the parasite materials available for the present study, our work and its outcomes overall represent a major advance. As discussed in the manuscript (lines 267 -270), future work should focus on resolving the sex chromosomes of *S. haematobium* using long-read data for pristine individual female and male worms as a foundation for explorations of sex-determining genes and sex- and developmentally-regulated gene transcription/expression.**

**2.7.** Gene models and annotations: what proportion of gene models were transferred by LiftOff (sic)?

**Response 2.7. This information is provided in the manuscript (lines 99 - 102). In total, 6227 genes were transferred using liftOver. We have now indicated the percentage (67.4%) that this number represents, and have corrected “liftOff” to “liftOver” (the name of this program) (line 399).**

**2.8.** Table 2 – can you add the accession numbers for the assemblies? The assembly and annotation of *S. mansoni* at least has changed over time, so would be good to be clear which is being analysed.

**Response 2.8. We downloaded genomic and proteomic data sets for all other schistosome species from WormBase Parasite (release WBPS15). This information has been added to Tables 1 (line 816) and 2 (line 821).**

**2.9.** Molecular variation: “distinct geographical locations”. Perhaps best describe where they are and how many worms.

**Response 2.9. This information is described in the Methods section and Supplementary Table 14: “… locations in Africa (Zambia, Senegal, Mauritius and Mali)…” (lines 311 - 316). Results (i.e. Figure 2 and Table 3) also provide relevant information and clearly indicate that individual male worms were used (lines 117 – 119).**

**2.10.** Following on from this, it seems like only 4 samples were compared. There are other WGS datasets available for *S. haematobium* from other populations (eg. PRJNA561522). Did you consider using some of these datasets?

**Response 2.10. Indeed individual male worms representing four distinct, distant geographic locations in Africa were compared. Our intent was to detect molecular ‘signatures’ in these worms to assess whether introgression events occur within the *S. haematobium* group. The results did reveal evidence for such events, which lays a foundation for detailed future investigations. Such a population genetic investigation to explore introgression cannot be conducted without chromosome level reference genomes for all recognised members of the *S. haematobium* group; would require adequate sample sizes (e.g., 40 to 50 individual male and female worms) per location; and is beyond the scope of the present study.**

**Regarding the suggested use of other *S. haematobium* data, the intent here was to compare data sets that were produced using the same experimental protocol. In our opinion and based on previous experiences, adding 200 whole-genome amplified *S. haematobium* samples (from Whatman FTA Cards) derived from miracidia from a single country (Nigeria) and under different experimental conditions would likely lead to challenges in the interpretation of findings. As our goal was to identify molecular evidence for introgression but not to undertake a population genetic investigation, we used four well-defined individual male worms from the SCAN collection (cf. Supplementary Table 14). We do not consider it appropriate to use unpublished data (recently made available) by another research group, as this group will likely want to pursue their own detailed analyses through a funded research project.**

**2.11.** “laboratory reference strain,” – where is the reference strain from?

**Response 2.11. We address this in the revised manuscript (lines 303-306); we refer to the reference strain, i.e., the BRI reference line which originates from Egypt (ref. 27).**

**2.12.** “Moderate or high impact” – there is huge difference in the potential effect of these two – perhaps best to state these numbers explicitly.

**Response 2.12. “Moderate” or “high impact” are defined categories for predicted coding effects obtained from the output from the program snpEff. These categories included the coding effects that are mentioned (“loss of a start codon, gain of a stop codon, or a non-synonymous alteration”). Numbers for individual categories are not listed in the snpEff output file, but we do include the merged VCF file for these analyses in the revised manuscript (Supplementary Data 1).**

**2.13.** “SNP-dense regions” – this description of the results is a little shallow. How are these regions defined, specifically? There is some implication that these represent introgression regions (at least in the discussion: “unique genomic signatures matching species other than S.haem…”) – if so, these should be more formally described.

**Response 2.13. We define the term “SNP-dense region” in the Methods (lines 464-466): “For each sample and each *S. haematobium* chromosome, the number of SNPs per 1 Mb non-overlapping region was determined, and regions with equal or more SNPs than 80% of all 1 Mb regions per chromosome (80th percentile) were selected as ‘SNP-dense’ locations”. In addition, we define ‘non-*S. haematobium* SNP signatures’ as “SNP-dense locations, for which > 20% of the 2000 nt sections (i.e. > 100 sections) matched those of a species other than *S. haematobium*.” (lines 474-476).**

**2.14.** Supplementary Fig. 1 is almost impossible to interpret. Could be better in landscape format perhaps?

**Response 2.14. A high-resolution image in landscape format accompanies the manuscript and has been uploaded separately.**

**2.15.** Variation in the transcriptome: There is a focus on the functional annotation/gene description of the top 1% of genes, but is there an actual enrichment of these gene sets in the top 1%, or are they just present?

**Response 2.15. We did not specifically investigate enrichment of particular functions of gene products encoded by the top 1% transcribed genes. We simply provide the functional annotations of these highly transcribed genes; some terms were common to genes transcribed in multiple stages/sexes. We consider our descriptions to be accurate and appropriate (lines 147 - 152).**

**2.16.** Differential isoform usage: I don’t think Fig. 3a communicates that there are distinct isoforms in two or more stages. Just simply looks like stage-specific expression.

**Response 2.16. We have removed the reference to Fig. 3a (heatmap) in this section. This heatmap represents the profiles for 13,941 transcripts (see Supplementary Table 4); it is not possible to present the cluster-isoform relationships for all individual genes in this image. To facilitate detailed information on isoforms for each gene and respective transcription clusters, we have added a gene identifier column to Supplementary Table 4.**

**2.17.** Differential isoform usage: The methods are very light on this, and in fact, don’t mention any specific analysis for quantifying differential isoform usage. Did you consider formally describing this – there are a number of tools available, and some precedent, for example, for *H. contortus* in Doyle et al 2020 CommBiol.

**Response 2.17. We inferred differential transcription using libraries derived from individual male and female worms (using six biological replicates each) using ‘Ballgown’. We have clarified in the Methods section (lines 489-493) that the Ballgown analysis was conducted at the transcript level (rather than the gene level), which allowed the inference of differentially transcribed isoforms between males and females (e.g., Fig. 4). For all developmental stages other than adults, biological replicates were not available (due to scarce materials), such that we were restricted to qualitative analyses and clustering of transcript isoforms according to transcription profiles. To clarify this point, we have altered the subtitle in the Results section (line 173).**

**2.18.** Genes with similar transcription profiles: “between 817 and 4301 transcripts” - which is it? How many in transcripts in total could be assigned to at least one of the clusters?

**Response 2.18. We have reformulated this sentence to clarify that the numbers are the range of the cluster sizes (lines 156 - 157). The sizes of individual clusters are given in Supplementary Table 4.**

**2.19.** Sex-linked transcription: “genes located on the ZW chromosome” – similar to the question above, did you define Z-specific and W-specific genes? Given you are talking about male and female specific transcription, this is pretty important. Similarly, genes present in the pseudo-autosomal regions of Z and W may also be interesting, particularly if you saw sex-specific expression. Did you see this at all?

**2.20.** “This scaffold represents a female-specific portion of the W chromosome” – can you check this and others with female WGS libraries? To what degree did you try and define W-specific sequences – this is not clear, but is relevant to some analyses and should be described.

**Responses 2.19 and 2.20. Our main goal was to assemble a chromosome-level genome for *S. haematobium*; we chose to do so independently (i.e. without using *S. mansoni* chromosomes to scaffold *S. haematobium* chromosomes). We achieved this goal and believe that the genome, as presented, is a very useful resource for the research community. Although the sex chromosomes of *S. haematobium* could not be unequivocally defined here using the parasite materials available for the present study, our work and its outcomes overall represent a major advance. As discussed (lines 267 - 270), future work should focus on resolving the sex chromosomes of *S. haematobium* using long-read data for pristine individual female and male worms (cf. response 2.6).**

**2.21.** Differential transcription in eggs: The results could use a single introductory sentence describing the rationale for doing this. You have it in the Discussion, however, I think it is needed here too. Similarly, there are clearly results presented in the Discussion that should be moved back into results, that might help round this section out a bit more.

**Response 2.21. We have addressed this and moved a section from the Discussion to the Results section (lines 214-223).**

**2.22.** “Well-defined laboratory (BRI) line” – perhaps remind the reader that it is Egyptian, as it becomes more relevant to describing African diversity. As a follow up, Biomedical Research Institute is never actually abbreviated as BRI, so that could perhaps be clarified.

**Response 2.22. We have added the abbreviation for Biomedical Research Institute upon first use in the manuscript (line 305) and have clarified that this laboratory strain originated from Egypt.**

**2.23.** Introgression / “unique genomic signatures” – this is potentially really interesting, esp. given the background, however, I found the analysis quite limited. There are a number of tools specifically focused on identifying introgression events based on phylogenetic incongruence, dating them etc. A lot in the *Helionchus* literature for example. The methods described here represent an interesting, perhaps novel approach to this question, but it is not clear how this approach compares to more established methods.

**Response 2.23. The accurate identification/characterisation of complex introgression events, as they likely occur within the *S. haematobium* group, and their source, will require chromosome-level reference genomes for all recognised members of *S. haematobium* group (that lack introgression and hybridisation signatures), so that phylogenetic trees can be constructed based on whole-genome alignments, and employing data for large numbers of samples from various (disparate) geographic location and species. Given the limited availability of such data sets and/or materials (including publicly available but yet unpublished data sets, the use of which we deem inappropriate), we elected to use four data sets representing *S. haematobium*. While this approach only permitted a guarded interpretation of the findings, we unequivocally showed the presence of introgression events in *S. haematobium*. Clearly, this evidence provides an exciting foundation for a detailed study of introgression in the future. Whether the methodology applied to studying introgression in *Helionchus* (sic) (we assume *Helianthus* was meant) is suitable for schistosomes would need to be assessed.**

**2.24.** “The proposal that variation in transcription levels in eggs…” – this is not clear to me. Who is making this proposal?

**2.25.** As a follow up, this sentence doesn’t make sense to me. States the differences in transcription between hamster and human relate to interaction between parasite and host and/or pathology – do you mean differences in transcription relate to differences in host-interactions and/or differences in pathology of infection? If so, this could be made clearer for a naïve reader.

**Responses 2.24. and 2.25. We have addressed this issue in the revised manuscript (lines 271 -275).** **Text now states “We propose that variation in transcription levels in eggs between hamster liver and human urine relate to differences in host-parasite relationship and to the ability of eggs to induce immunopathological changes and disease (which is pronounced in humans, but not in the hamster), including the presence of *S. mansoni* homologs of IPSE/*alpha-1* [40, 41], *kappa-5* [42] and ribonuclease *omega*-*1* [43].”**

**2.26.** AlphaFold results should be in the results, and results shown somewhere.

**Response 2.26. We have moved this section to the Results (lines 218-223), and have added a Supplementary Table with the results from the AlphaFold analysis (Supplementary Table 13).**

**2.27.** “From a technical perspective, the short-read sequencing…” – it reads like you have done this for the first time, which is not technically correct. I suggest tone this down.

**Response 2.27. We have amended this section in the Discussion (lines 287 - 291).**

**2.28.** For all sequencing, including the use of older, publicly available data – was BRI used? Not really clear.

**Response 2.28. For all sequencing, the BRI laboratory line (i.e. originally from Egypt) was used, with the exception of the four WGS samples from distinct geographic locations in Africa (Zambia, Senegal, Mauritius and Mali) and the eggs from urine samples (Cameroon). The Shae.V3 assembly represents this BRI laboratory line (Egyptian strain). We have clarified this in the revised manuscript.**

**2.29.** HiC – what method was used? Was there any manual curation of the assembly based on the contact map information?

**Response 2.29. 3D-DNA software (ref. 68) was used to scaffold the contigs. Further manual curation was done using contact map information and using Juicer software (ref. 67). The text has been amended in the Materials and Methods (lines 342 – 344).**

**2.30.** Synteny analysis – “linking single-copy orthologs in a pairwise manner” – what does this mean exactly?

**Response 2.30. Single-copy orthologs were used to link genomic scaffolds/chromosomes for each species pair (Shae.V3 and one other genome, respectively) as shown in Figures 1a-d. We have changed the text to “for each species-pair” (line 356), to clarify this aspect.**

**2.31.** Great that the code for the analysis and recreating of the figures is made available on GitLab, however, I can’t seem to access it. Could the authors please check it is visible.

**Response 2.31.** **Herewith we make the code publicly available *via*** [**https://gitlab.unimelb.edu.au/bioscience/s_haematobium_v3**](https://gitlab.unimelb.edu.au/bioscience/s_haematobium_v3)**.**

**2.32.** Data availability. I can’t see the V3 genome in ENA yet under the project ID provided. Can you confirm the genome and annotation have been submitted somewhere?

**Response 2.32. The genome assembly has been submitted as an update to the existing genome assembly under the NCBI BioProject ID PRJNA78265 and the GenBank assembly accession GCA_000699445.3. The DDBJ/ENA/GenBank accession is AMPZ00000000. The version described in this paper is AMPZ03000000, and will be made publicly available following acceptance of the present manuscript. For the purpose of reviewing this submission, the genome and associated GFF file can be accessed *via* the following online repository: https://unimelbcloud-my.sharepoint.com/:f:/g/personal/nyoung_unimelb_edu_au/EjU_6mzaR4pMpnaUexWuiAsBPD6uhY2yBF6ovi4ZmNW6KQ?e=ahMDNj (password: Shaematobium).**

**Reviewer #3:**

**3.1.** While the assembly and annotation are notable improvements over previous versions, the manuscript felt like an announcement of an incremental update and presentation of a resource. I finished reading the manuscript and wondered what major new understanding of *S. haematobium* biology was discovered. Analysis of the RNA-Seq data is a major feature of the results, and it is difficult to tease out what are the novel discoveries. For example, “[i]n the sporocyst stage, unique transcripts encoded… venom allergen-like” [proteins].” However, in Young et al. 2012, venom allergen-like proteins “ES proteins in the egg.” And, in Figure 3 of the earlier paper, these proteins are highly expressed in the egg compared to adults. The findings of the submitted manuscript are not necessarily incongruent with the earlier paper, but proper context is required.

**3.2.** I consider it important that the authors present what is novel about the current study, especially around the transcriptomics.

**Responses 3.1. and 3.2. The intent of the present study was to provide a substantially enhanced resource to the research community. In addition to achieving contiguity of the genome assembly, facilitated by using novel and emerging sequencing technologies and data sets (including chromatin conformation capture and Oxford Nanopore long-read sequencing), the present work also provides completely novel RNA-Seq data sets for key developmental stages (sporocyst and cercariae) as well as a data set from *S. haematobium* eggs from the human host; none of these data have been available or accessible until now.**

**We demonstrate the usefulness and utility of this new resource (the genome) to the research community by: (i) identifying novel genes that can now be investigated in detail (lines 110-112 and 218-223); (ii) characterising transcripts linked to particular developmental stages (lines 173 - 212); and (iii) providing first evidence of variation in the host-parasite relationship and the ability of eggs to induce disease in particular host species (lines 214 - 231).**

**We believe that the findings and hypotheses/proposals will stimulate significant future investigations of numerous aspects of *S. haematobium* biology by the research community. Regarding the presence/absence of venom allergen-like protein-encoding transcripts from distinct stages of *S. haematobium*, we wish to indicate that this protein family is relatively large (containing ≥ 28 members in *S. mansoni*; e.g., https://www.ncbi.nlm.nih.gov/pmc/articles/PMC3435950/) and as such, the unique transcripts found in the sporocyst and cercarial stages (for which no RNA-Seq data had been available for *S. haematobium*) do not represent the same genes identified in earlier studies. In summary, we believe that we have provided a clear and sound description, interpretation and discussion of key findings. We are satisfied with our discussion. Based on point 2.1, we have enhanced the description of findings in the Abstract of this manuscript (lines 20 - 27).**

**3.3.** The section of molecular variation between four locations was purely descriptive. In the Discussion, the authors call the observed variation ‘substantial’, however, they give no context with respect to other *Schistosoma* species. Further, I would like them to consider the work of Gower et al 2013 (http://dx.doi.org/10.1016/j.actatropica.2012.09.014) which investigated *S. haematobium* population structure.

**Response 3.3 We agree that our findings of molecular variation for four individual worms from four distinct locations are descriptive. Defining population structures, as the reviewer suggests, would require chromosome-level references for all *S. haematobium* group species and a large number of samples (individual worms representing each sex) from individual locations. Given the limited availability of such published material and such published data sets, our objective here was to identify molecular ‘signatures’ in each of four isolates investigated herein, to assess the occurrence of introgression within *S. haematobium*. This work provides a solid foundation for a large-scale population genetic/genomic investigations in the future, provided that chromosome-level genomes are produced for individual members of the *S. haematobium* group.**

**3.4.** The population genomic analysis is presented as an UpSet plot and piecharts. This lacks the sophistication that has become standard in population genomics. I strongly recommend that the authors use the program STRUCTURE (https://web.stanford.edu/group/pritchardlab/structure.html) or another robust approach.

**Response 3.4. The present study is not a population genetic or population genomic study. We used four samples (individual males) from four distinct geographic locations, not entire populations representing these locations. Accordingly, we restricted our analysis to exploring molecular variation among those four samples with respect to the new, chromosome-level reference genome (representing the BRI laboratory strain, which is widely used for experimentation). We discuss the implications of this new resource for systematic, large-scale population genomic/genetic explorations of *S. haematobium* in the future (lines 287-291). The use of the program STRUCTURE will be very useful for this purpose; we thank the reviewer for their suggestions.**

**3.5.** Differential gene expression features prominently in the submitted manuscript. Not all the transcriptomics data are available in triplicate. This means it is not possible to conduct statistically robust comparisons (doi.org/10.1261/rna.053959.115). Either the authors need to generate appropriate replication datasets for all stages under consideration or restrict their analysis to only those datasets done in triplicate.

**Response 3.5. Differential transcription analysis using ‘Ballgown’ was done for the male-female comparisons (using six biological replicates each; see Methods; lines 362-363 and 489-490 and the new Supplementary Table 1). We did not infer (quantitative) differential transcription for samples without replicates. In the absence of replicates, we conducted qualitative analyses of transcription, and described the presence/absence of genes/transcripts among stages and/or predominant profiles. These data sets were derived from pooled worms/eggs, and as such, the transcription profiles recorded are essentially normalised (among individuals with these pools) and reliably represent these developmental stages.**

**3.6.** The SRA data for this paper remain ‘not public’. When released will the data include the sequencing runs for the four populations from geographical locations? I ask because, in the methods, I could not find specific run accessions for these in the papers.

**Response 3.6. All data produced for this study have been deposited to the Sequence Read Archive under accession numbers SRR15400745 - SRR15400772, including the four WGS data sets representing the four geographic locations in Africa (accessions SRR15400745 and SRR15400770 - SRR15400772). These data would be made publicly available upon publication of the manuscript. We have included Supplementary Table 1 that describes all data sets produced for this study. All other data produced in previous studies, and used here, are acknowledged through the citation of respective articles and accession numbers/codes.**

**_______________________________**

**CONCLUDING REMARKS**

**We are grateful for the thorough and constructive reports on our manuscript. We have addressed all of the reviewers’ comments and have modified manuscript (highlighted in yellow) in accordance with the present rejoinder (line numbers). In our opinion, the R1 manuscript now meets the high standard required for publication in *PLoS Pathogens*. We look forward to the final decision on our manuscript.**

Yours sincerely,

Neil D. Young and Robin B. Gasser

On behalf of all authors.

The University of Melbourne | Corner Flemington Road & Park Drive, Parkville, Victoria 3010, Australia | [nyoung@unimelb.edu.au](mailto:nyoung@unimelb.edu.au); [robinbg@unimelb.edu.au](mailto:robinbg@unimelb.edu.au)
